# Identification of key programmed cell death genes for predicting prognosis and treatment sensitivity in colorectal cancer

**DOI:** 10.3389/fonc.2024.1483987

**Published:** 2024-11-13

**Authors:** Jian-ying Ma, Yi-xian Wang, Zhen-yu Zhao, Zhen-yu Xiong, Zi-long Zhang, Jun Cai, Jia-wei Guo

**Affiliations:** ^1^ Department of Gastrointestinal Surgery, Jingzhou Hospital Affiliated to Yangtze University, Jingzhou, China; ^2^ Department of Interventional, Jingzhou Hospital Affiliated to Yangtze University, Jingzhou, China; ^3^ Department of Immunology, School of Medicine, Yangtze University, Jingzhou, China; ^4^ Department of Pharmacology, School of Medicine, Yangtze University, Jingzhou, China; ^5^ Department of Oncology, First Affiliated Hospital of Yangtze University, Jingzhou, China

**Keywords:** colorectal cancer, programmed cell death, prediction model, immunotherapy, drug sensitivity

## Abstract

Colorectal cancer (CRC) ranks third in global incidence and second in mortality. However, a comprehensive predictive model for CRC prognosis, immunotherapy response, and drug sensitivity is still lacking. Various types of programmed cell death (PCD) are crucial for cancer occurrence, progression, and treatment, indicating their potential as valuable predictors. Fourteen PCD genes were collected and subjected to dimensionality reduction using regression methods to identify key hub genes. Predictive models were constructed and validated based on bulk transcriptomes and single-cell transcriptomes. Furthermore, the tumor microenvironment, immunotherapy response, and drug sensitivity profiles among patients with CRC were explored and stratified by risk. A risk score incorporating the PCD genes FABP4, AQP8, and NAT1 was developed and validated across four independent datasets. Patients with CRC who had a high-risk score exhibited a poorer prognosis. Unsupervised clustering algorithms were used to identify two molecular subtypes of CRC with distinct features. The risk score was combined with the clinical features to create a nomogram model with superior predictive performance. Additionally, patients with high-risk scores exhibited decreased immune cell infiltration, higher stromal scores, and reduced responsiveness to immunotherapy and first-line clinical drugs compared with low-risk patients. Furthermore, the top ten non-clinical first-line drugs for treating CRC were selected based on their predicted IC50 values. Our results indicate the efficacy of the model and its potential value in predicting prognosis, response to immunotherapy, and sensitivity to different drugs in patients with CRC.

## 1 Introduction

Colorectal cancer (CRC) is one of the most common malignancies, ranking third in incidence and second in mortality worldwide (1). Various treatment modalities are available for CRC, including chemotherapy and immunotherapy (2). Unfortunately, clinical outcomes remain poor owing to the heterogeneity of CRC, genetic features, and distinct risk factors (3, 4). Approximately 25% of patients with CRC are diagnosed at an advanced stage, and 25–50% of patients diagnosed at an early stage eventually go on to develop metastatic disease (5–7). The 5-year survival rate for individuals with limited metastatic lesions remains at 40% following surgical resection and systemic therapy, whereas for those with advanced metastatic CRC, the survival rate drops to only 20% (8–11). Considering the low survival rate of patients with CRC, there is an urgent need to identify an accurate classification of CRC that can better predict patient outcomes, immunotherapy, and chemotherapy responses, ultimately guiding personalized treatment strategies and improving patient prognosis.

Programmed cell death (PCD) plays a crucial role in cancer occurrence and treatment (12). PCD driven by various complex mechanisms includes apoptosis, pyroptosis, ferroptosis, autophagy, necroptosis, cuproptosis, anoikis, parthanatos, entotic cell death, NETosis, lysosome-dependent cell death, alkaliptosis, oxeiptosis, and disulfidptosis (13–16). We selected these 14 PCD types to comprehensively cover the diverse mechanisms relevant to CRC while ensuring specificity. This number reflects an optimal balance, capturing all major PCD pathways with distinct molecular signatures, based on an exhaustive review of literature and databases, without introducing redundancy or lesser-studied types that could complicate analysis. Apoptosis critically regulates CRC progression by either inhibiting tumor growth when activated or promoting tumor survival and metastasis when suppressed. Studies have shown that targeting apoptotic pathways, such as enhancing microRNA-induced apoptosis, can sensitize CRC cells to treatment and inhibit tumor progression (17, 18). Pyroptosis is crucial in suppressing CRC progression by activating immune responses. Recent studies emphasize that pyroptosis-inducing therapies, like quercetin or mitochondria-targeted photodynamic therapy, not only inhibit tumor growth but also enhance anti-tumor immunity, offering promising avenues for CRC treatment (19, 20). Recent advances in ferroptosis research have highlighted its role in CRC progression and treatment resistance. Studies demonstrate that targeting ferroptosis regulators, such as SLC7A11 or GPX4, may enhance therapeutic efficacy by overcoming chemoresistance and inducing cancer cell death (21, 22). Autophagy plays a dual role in CRC, influencing both tumor suppression and progression. Targeting autophagy regulators, such as lncRNA GAS5 and ATG9A, holds promise for improving chemotherapy response and modifying the tumor microenvironment (TME) (23, 24). Necroptosis has been shown to play a pivotal role in CRC by driving inflammatory processes and facilitating cancer cell death. By modulating necroptotic pathways, particularly through the RIPK1/RIPK3/MLKL signaling cascade, new therapeutic strategies can potentially suppress tumor progression and improve treatment outcomes in CRC (25). Cuproptosis, a copper-dependent cell death mechanism, is triggered by mitochondrial dysfunction and protein aggregation. In CRC, targeting cuproptosis pathways has shown potential for tumor suppression and improving immune response (26, 27). Anoikis, induced by cell detachment from the extracellular matrix, is crucial in preventing metastasis. In CRC, KHK-A-mediated phosphorylation of PKM2 enables anoikis resistance and promotes metastasis (28). Parthanatos, driven by PARP-1 overactivation, contributes to tumor progression through DNA damage and mitochondrial dysfunction. Its role in modulating the TME has been recently highlighted (29, 30). Entotic cell death, initiated by loss of cell adhesion, involves neighboring cells engulfing each other. TRAIL signaling promotes entosis, potentially contributing to CRC suppression (31, 32). NETosis, a form of cell death in neutrophils, involves the release of neutrophil extracellular traps to capture pathogens, driven by ROS and histone modification. Recent advances highlight the role of NETosis in CRC, contributing to tumor progression by promoting an inflammatory microenvironment (33). Lysosome-dependent cell death (LCD) is driven by lysosomal membrane permeabilization, releasing cathepsins and causing cellular damage. Existing studies indicate that modulating lysosomal function in CRC could enhance tumor sensitivity to LCD, providing a strategy to overcome drug resistance (34, 35). Alkaliptosis, a pH-dependent cell death, involves intracellular alkalinization and lysosomal dysfunction. It shows potential as a therapeutic approach in CRC by disrupting tumor cell survival (36, 37). Oxeiptosis, a ROS-dependent cell death via the KEAP1-PGAM5-AIFM1 axis, shows potential as a tumor-suppressive mechanism in CRC, with sanguinarine being a key inducer (38). Disulfidptosis, driven by actin cytoskeleton collapse via disulfide bond formation, shows potential in CRC for prognosis prediction and therapeutic advancements (15, 39, 40). Notably, drugs targeting PCD pathways have already entered clinical application, offering novel therapeutic potential for CRC treatment. For instance, a BCL-2 inhibitor approved by the FDA has been shown to exert protective effects against lymphoma through apoptosis (41). gasdermin E mediated pyroptosis is intimately linked to the enhancement of anti-tumor immunity (42). Ferroptosis-targeted drugs or genetic manipulations can help against chemotherapy resistance (43).

Various PCD types play crucial roles in the progression and treatment of malignant tumors (12, 44). However, the integration of various PCD-related genes to identify key hub genes that significantly impact CRC prognosis has yet to be fully elucidated. To address this, we employed Cox proportional hazards and LASSO regression to select PCD-related genes through dimensionality reduction. The Cox model allowed us to identify genes associated with CRC prognosis by estimating hazard ratios, making it effective for survival analysis. LASSO regression was applied to manage the high-dimensional genetic data, selecting the most influential genes while reducing overfitting. By integrating these methods, we identified crucial PCD-related genes and constructed a robust model to predict the prognosis of CRC patients, as well as their response to immunotherapy and drug sensitivity, ensuring the clinical relevance and reliability of our findings.

## 2 Materials and methods

### 2.1 Data collection

The gene sets associated with 14 types of PCD were collected from the GSEA database, FerrDB database, and literature sources (45). The specific contents of the gene sets are provided in the supplementary materials and can also be accessed through the aforementioned databases using the gene set names. Following consolidation and deduplication, 1,267 PCD genes were included for subsequent analysis (**Supplementary Table S1**). The datasets used for analysis include TCGA-CRC, GSE39582, GSE29621, GSE17536, GSE38832, GSE161277, GSE78220, and IMvigor210.

### 2.2 Identification of differentially expressed genes

Differential analysis was utilized to identify DEGs between tumor and normal tissues, with criteria set as |log2 fold change| > 1.5 and adjusted P < 0.05. Similarly, for DEGs between the low- and high-risk groups, the criteria were |log2 fold change| > 1 and adjusted P < 0.05.

### 2.3 Construction and validation of prognostic PCD-related gene signature

This process employs a stepwise regression strategy, beginning with univariate Cox regression to identify PCD-related genes significantly associated with survival, followed by LASSO Cox regression for dimensionality reduction, and culminating in multivariate Cox regression to develop the risk score. The risk score for each patient was calculated using the following formula:


Risk score=∑ βi*Ei

This formula incorporated the risk coefficients ( βi ) and the expression levels of individual genes ( Ei ). Subsequently, the t-distributed stochastic neighbor embedding (t-SNE) was utilized for clustering analysis of high- and low-risk groups. Kaplan-Meier analysis was then performed to explore the relationship between overall survival (OS) time and the calculated risk score.

### 2.4 Unsupervised clustering of PCD-related gene signature

Utilizing the PCD-related gene signature, we performed consensus clustering (CC) analysis to uncover previously unidentified subtypes of CRC. Additionally, we employed the t-SNE for clustering analysis of these CRC subtypes.

### 2.5 Establishment of the nomogram

Univariate Cox regression analysis was conducted to evaluate the prognostic value of various clinical characteristics and risk score. multivariate Cox regression analysis was utilized to identify prognostic factors associated with OS, which served as the foundation for developing a prognostic nomogram. The predictive efficiency of the nomogram was evaluated using calibration curves and receiver operating characteristic (ROC) curves.

### 2.6 Tumor microenvironment

Algorithms such as XCELL, TIMER, QUANTISEQ, MCPCOUNTER, ESTIMATE, EPIC, CIBERSORT, and ssGSEA were utilized to evaluate TME cell infiltration in patients with CRC.

### 2.7 Prediction of therapeutic sensitivity in patients with different risk scores

The association between the risk score, immune treatment response, and the IC50 values of 198 chemotherapy and targeted therapy drugs was investigated. The TIDE algorithm was applied to predict patients’ potential response to immunotherapy, while the oncopredict package was employed to calculate IC50 values for each patient across the 198 drugs.

### 2.8 Cell lines and culture

The LOVO, SW480 and NCM460 cells were purchased from the Type Culture Collection of the Chinese Academy of Sciences (Shanghai, China). All used cell lines were maintained in Dulbecco’s Modified Eagle Medium supplemented with 10% fetal bovine serum and 1% penicillin-streptomycin and grown in a humidified atmosphere containing 5% CO2 at 37°C. All cell lines were authenticated using short tandem repeat (STR) genotyping and tested negative for Mycoplasma.

### 2.9 Terminal deoxynucleotidyl transferase deoxyuridine triphosphate nick end labeling staining

Apoptosis analysis of CRC cells was performed using TUNEL staining (Millipore, Billerica, MA, USA) following the manufacturer’s instructions. Briefly, the cells were seeded on coverslips in 24-well plates at a density of 2×105 cells/well. The cell slides were then fixed in 4% paraformaldehyde for 60 min, washed with phosphate-buffered saline (PBS), permeabilized using 0.1% Triton X-100 for 2 min on ice, and stained with TUNEL detection liquid for 1 h at room temperature in the dark. Subsequently, the cells were washed twice with PBS and stained with DAPI to visualize the cell nuclei for 10 min at room temperature to visualize the cell nuclei. Fluorescent signals were visualized using a laser scanning confocal microscope (LSM800; Zeiss, Jena, Germany).

### 2.10 Annexin V-FITC/PI apoptosis assay

The number of apoptotic CRC cells was determined using an Annexin V-FITC/propidium iodide (PI) assay kit (BD Biosciences, New Jersey, USA) according to the manufacturer’s instructions. In brief, the collected CRC cells were washed with cold PBS and resuspended in 500 μl 1× binding buffer. Subsequently, 5 µL of annexin-V-FITC and 5 µL of PI were added to 200 µL of each sample, gently vortexed, and incubated in the dark for 15 min at room temperature. Finally, the number of stained cells was detected using a flow cytometer (BD Biosciences, San Jose, CA).

### 2.11 Plasmid construction and transfection

Human full-length NAT1, amplified from the human cDNA library, was cloned into the pEGFP-N1 vector to overexpress NAT1. Plasmid transfection was performed using Lipofectamine 2000 reagent (Invitrogen) according to the manufacturer’s instructions.

### 2.12 Quantitative real-time PCR and western blotting

The mRNA levels were analyzed by qPCR assay as previously described (46–48). Briefly, total RNA was extracted from cells using TriPure Isolation Reagent (Roche, Basel, Switzerland), following the manufacturer’s instructions. Subsequently, 2 µg of total RNA was reverse-transcribed into cDNA using a Transcriptor First Strand cDNA Synthesis Kit (Roche). Real-time PCR was performed on a MyiQ Single Color Real-time PCR Detection System (Bio-Rad Laboratories, Hercules, CA, USA) using the SYBR Green PCR Master Mix (Bio-Rad Laboratories). The relative mRNA expression levels were normalized to that of β-actin mRNA using the comparative 2–ΔΔCT method. Primer sequences used for the indicated genes are listed in **Table 1**.

**Table 1:** Primer sequences used for real-time PCR analysis.

Gene	Forward primer (5′-3′)	Reverse primer (5′-3′)
NAT1 (human)	GGGAGGGTATGTTTACAGCAC	ACATCTGGTATGAGCGTCCAA
AQP8 (human)	CCATGTGTGAGCCTGAATTTGG	ACCCGATGAAGATGAAGAGAGC
FABP4 (human)	ACTGGGCCAGGAATTTGACG	CTCGTGGAAGTGACGCCTT
β-actin (human)	GCTTCTCCTTAATGTCACGC	CCCACACTGTGCCCATCTAC

For protein extraction, cell samples were homogenized and lysed in radioimmunoprecipitation assay (RIPA) lysis buffer containing a protease inhibitor cocktail (Roche) and a phosphatase inhibitor (Roche). Protein concentrations were determined using a bicinchoninic acid (BCA) assay kit (Thermo Fisher Scientific, Waltham, MA, USA). Equal amounts of collected protein (50 µg) were then separated by sodium dodecyl sulfate-polyacrylamide gel electrophoresis (SDS-PAGE) on 8–12% gels and transferred onto polyvinylidenefluoride membranes (Millipore) via electroblotting. Subsequently, the membranes were blocked with 5% nonfat milk in 1× tris-buffered saline with tween 20 (TBST) at room temperature and incubated with primary antibodies overnight at 4°C (primary antibodies listed in **Table 2**). After washing with TBST thrice, the membranes were incubated with horseradish peroxidase-conjugated secondary antibodies for 1 h at room temperature. Membranes were visualized using enhanced chemiluminescence reagent (Thermo Fisher Scientific) and immunoblotting images were captured using the ChemiDoc MP Imaging System (Bio-Rad). The housekeeping gene *β-actin* was used as the internal control, and the gray value of each band was quantitatively analyzed using ImageJ software (National Institute of Health, Bethesda, MD, USA).

**Table 2:** Antibodies used for western blot analyses.

Antibodies	Source	Cat NO.	Dilution
β-actin	Proteintech	20536-1-AP	1:2000
BAX	Proteintech	50599-2-Ig	1:2000
Cleaved Caspase3	Cell Signaling Technology	9664	1:1000
Caspase3	Cell Signaling Technology	9662	1:1000

### 2.13 Statistical analysis

All statistical analyses were performed using R (version 4.3.1). Continuous variables were compared using the Wilcoxon rank-sum test, appropriate for non-normally distributed data such as gene expression levels. Categorical variables were analyzed with the Chi-square test. For comparisons involving more than two groups, the Kruskal-Wallis test was used as a non-parametric alternative to ANOVA, suitable for non-normally distributed data, including gene expression and other derived metrics. Spearman’s rank correlation was applied to assess associations between variables, given its robustness in handling non-linear and non-parametric data. Statistical significance was defined as p < 0.05.

## 3 Results

### 3.1 Workflow of this study

**Figure 1:**
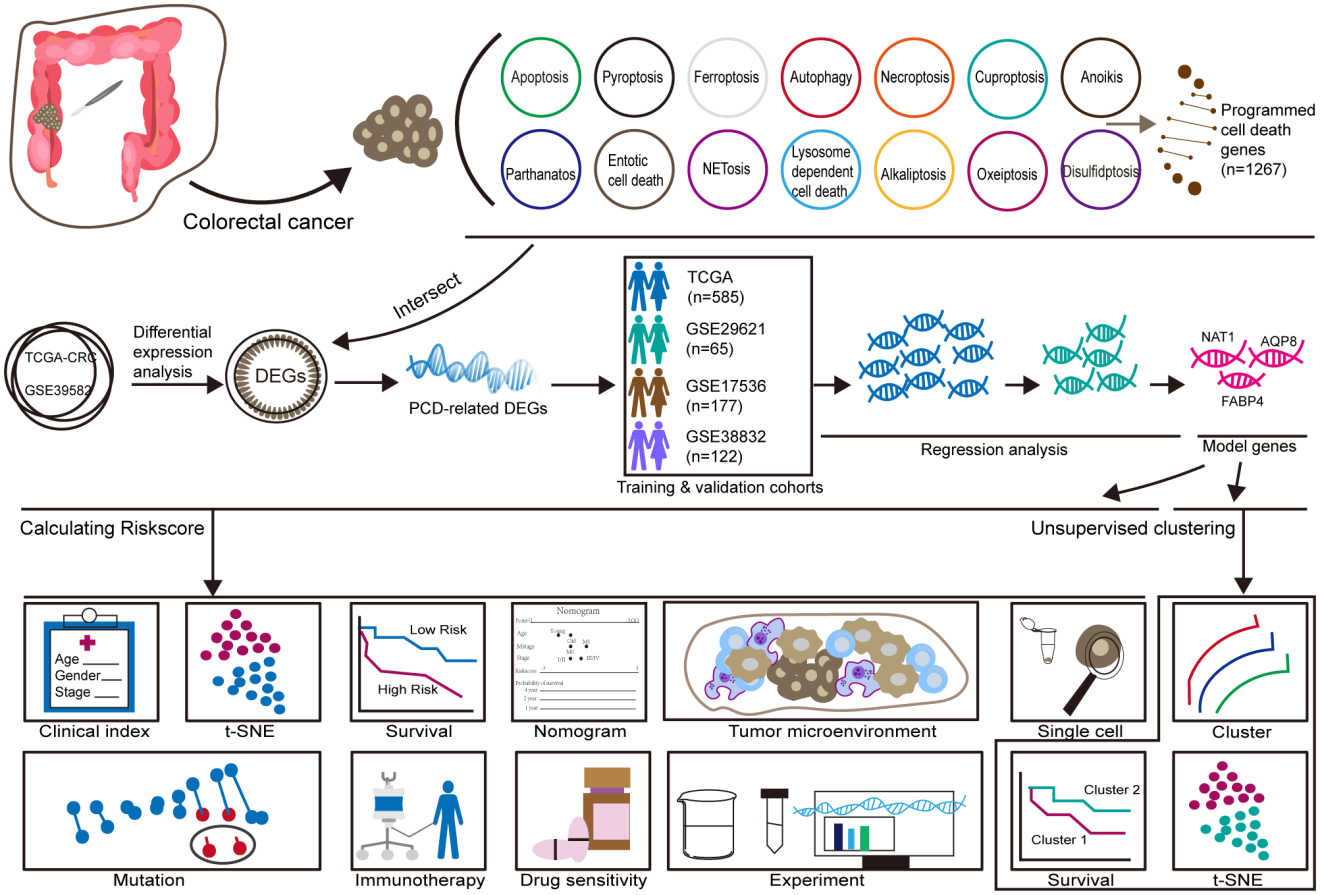
Workflow.

The training cohort included 585 patients with CRC from the TCGA database, while the validation cohort consisted of 65 patients from GSE29621, 177 patients from GSE17536, and 122 patients from GSE38832. A flowchart summarizing the study design is provided in **Figure 1**.

### 3.2 Identification of PCD-related DEGs

**Figure 2:**
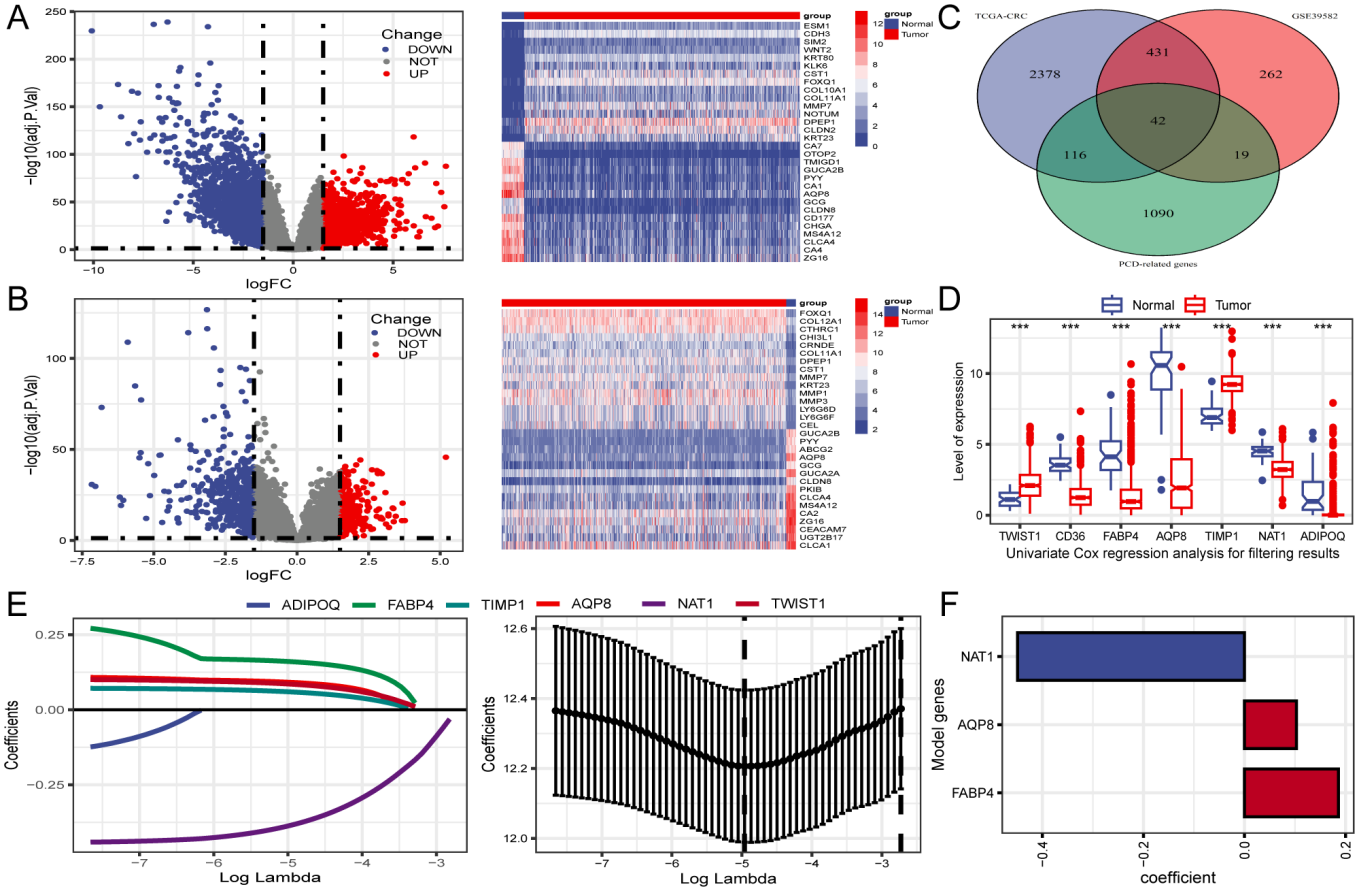
Identification of differentially expressed genes (DEGs) related to PCD and construction of prognostic gene signature. **(A, B)** Volcano plot and heatmap of DEGs between CRC and normal tissues in TCGA and GSE39582. **(C)** Venn diagram reveals 42 PCD-related DEGs obtained from the intersection. **(D)** The expression profiles of seven genes associated with prognosis identified through univariate Cox regression analysis. **(E)** LASSO regression employed for screening of univariate COX regression results. **(F)** A final selection of 3 genes associated with the prognosis of patients with CRC was made through multivariate COX regression analysis to create a signature. ***P < 0.001.

Differential expression analysis identified 2,967 DEGs from the TCGA-CRC dataset (**Figure 2A**). To improve accuracy, the same analysis was performed on GSE39582 (**Figure 2B**). We subsequently identified the intersection of DEGs from both cohorts with 1,267 PCD-related genes, yielding 42 differentially expressed PCD-related genes, as illustrated in the Venn diagram (**Figure 2C**).

### 3.3 Construction of a PCD-related gene signature

**Figure 3:**
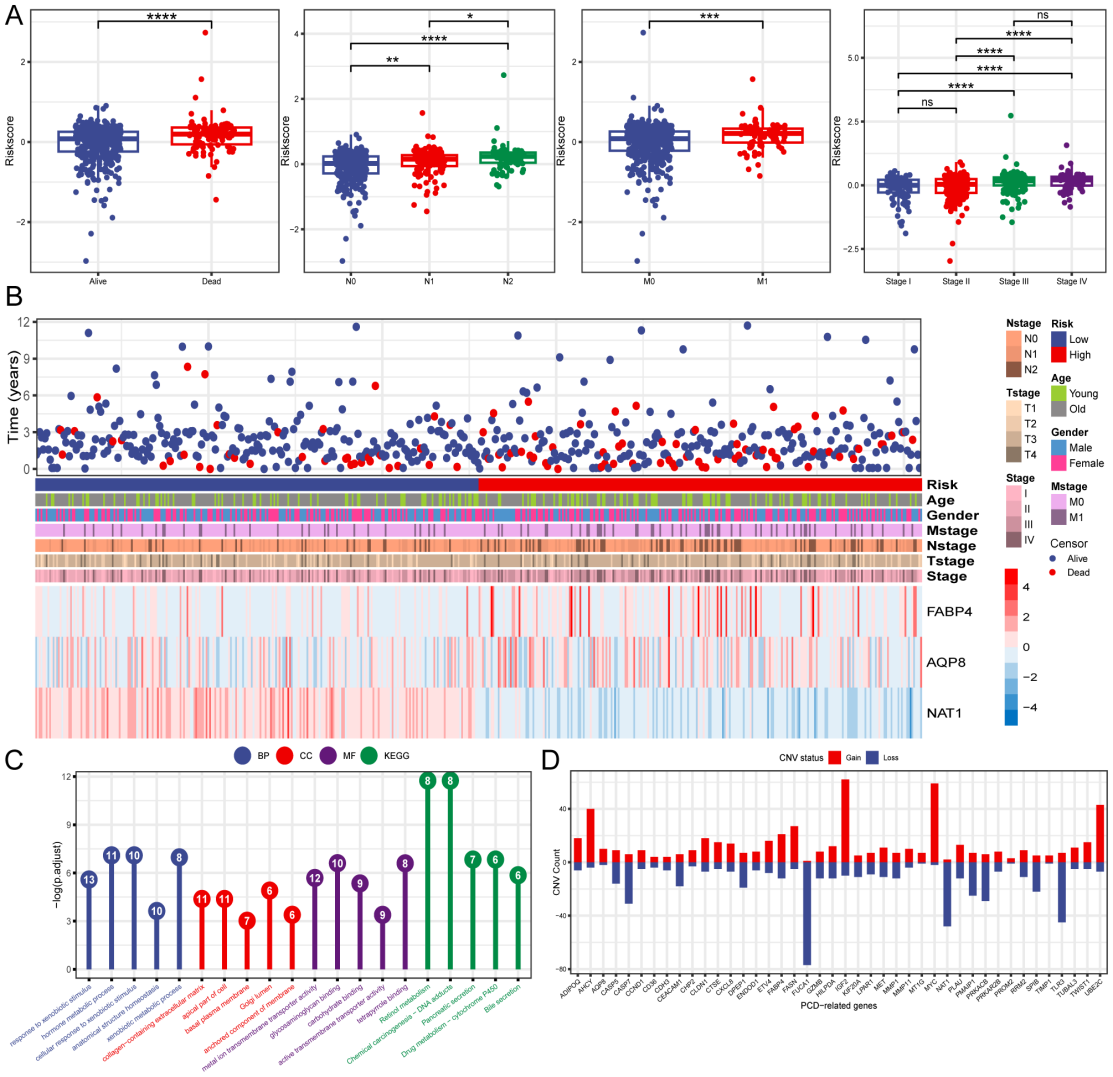
Clinical relevance and functional analysis of the constructed gene signature. **(A)** Box plots depicting the association between risk score and clinical characteristics. **(B)** Heatmap regarding the expression of the three model genes and the patient’s features. **(C)** Enrichment analysis the DEGs in high- and low-risk groups. **(D)** CNV status of 42 PCD-related DEGs. *P < 0.05; **P < 0.01; ***P < 0.001; ****P < 0.0001.

Three PCD-related DEGs (FABP4, AQP8, and NAT1) with prognostic significance were identified using univariate, Lasso-Cox, and multivariate Cox regression analyses (**Figures 2D–F**). Subsequently, Kaplan-Meier analysis was performed to determine the prognostic value of FABP4, AQP8, and NAT1, with OS as the outcome metric. Patients with CRC with high NAT1 and AQP8 expression showed better OS, whereas high FABP4 expression significantly correlated with shorter OS (**Supplementary Figure S1**). The risk score for each patient was calculated using the formula: risk score = (0.186 × FABP4) + (0.103 × AQP8) + (-0.449 × NAT1). We investigated the associations between risk scores and diverse clinical characteristics, including survival status, clinical stage, N stage, and M stage. Poor clinical outcomes were associated with higher risk scores (**Figures 3A, B**). Patients with CRC were stratified into high- and low-risk groups based on the median risk score to evaluate functional differences between the two groups. GO and KEGG analyses were applied to identify the biological functions and pathways of the 150 DEGs in the high- and low-risk groups (**Supplementary Table S2**). We focused on the top five enrichment results from both GO and KEGG and discovered that the biological processes were involved in metabolic processes. In addition, the KEGG analysis of the DEGs indicated that retinol metabolism and drug metabolism-cytochrome P450 were among the most significantly enriched pathways (**Figure 3C**). Furthermore, a close relationship between 42 PCD-related DEGs and CNV was explored in the TCGA-CRC cohort. Notably, AQP8 and FABP4 were frequently expressed, whereas NAT1 was occasionally expressed in CRC (**Figure 3D**).

### 3.4 Internal training and external validation of the PCD-related gene signature

**Figure 4:**
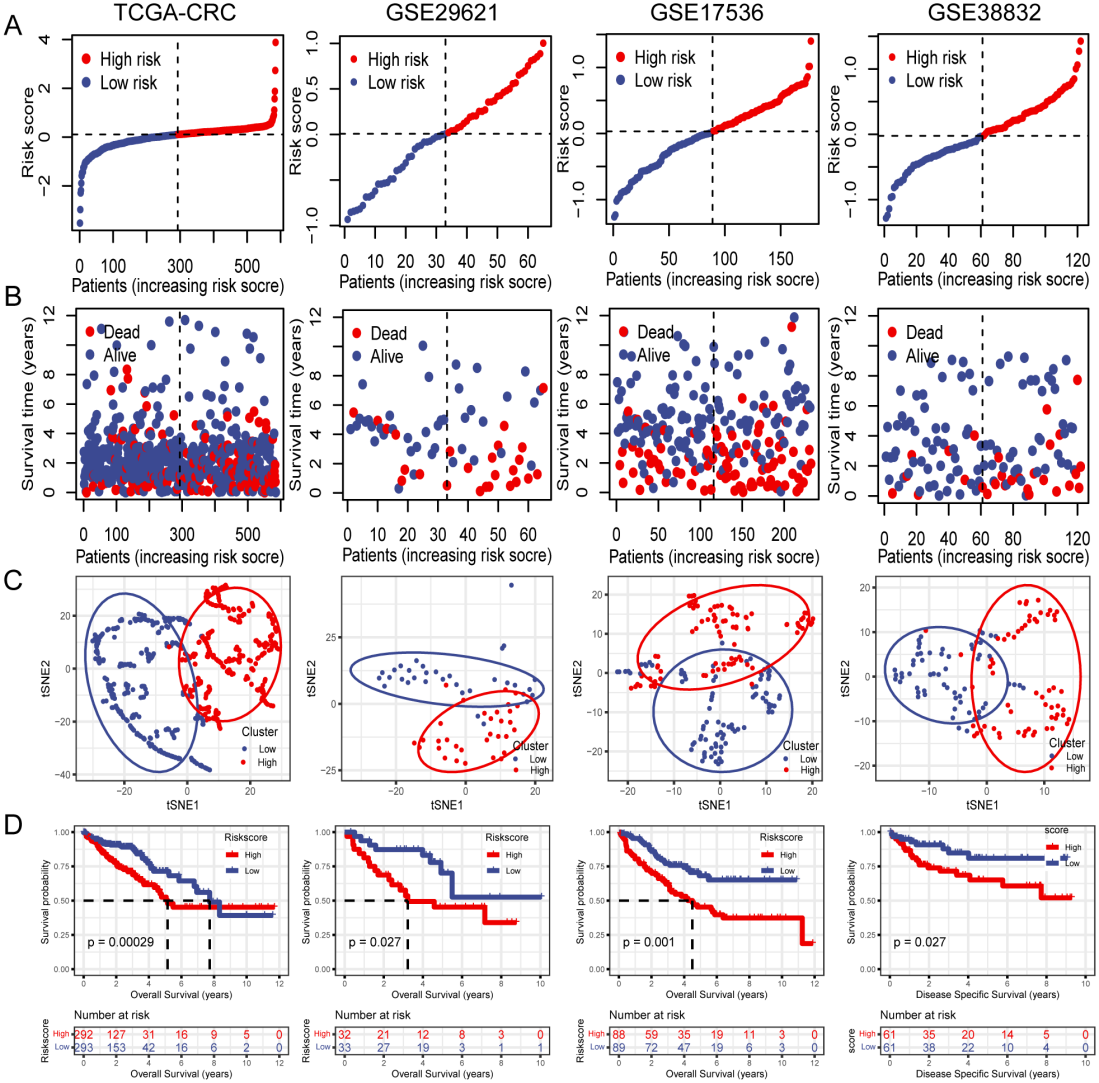
Internal training and external validation of the gene signature prediction model. **(A)** Distribution of risk score between low- and high-risk groups. **(B)** Survival status of patients with CRC in the low- and high-risk groups. **(C)** t-SNE analysis plot based on the risk group. **(D)** The differences between OS and DSS in different risk groups.

Subsequently, OS rates were compared between patients with high- and low-risk scores in the TCGA training cohort. The OS rate was significantly lower in patients with CRC who had high-risk scores than in those with low-risk scores (**Figures 4A, B**). Additionally, t-SNE analysis revealed that the established risk score was suitable for classification (**Figure 4C**). There was a significant difference in the OS between the two groups according to the risk score. Patients in the low-risk group had a better OS than those in the high-risk group (**Figure 4D**). The GSE29621, GSE17536, and GSE38832 datasets were used as validation cohorts. Patients from these validation cohorts were divided into high- and low-risk groups based on the median risk scores. Consistent with the results of the training cohort, the survival rates, OS, and Disease Specific Survival (DSS) in the validation cohort were lower in the high-risk group than those in the low-risk group (**Figures 4A–D**).

### 3.5 Unsupervised clustering of PCD-related gene signature

**Figure 5:**
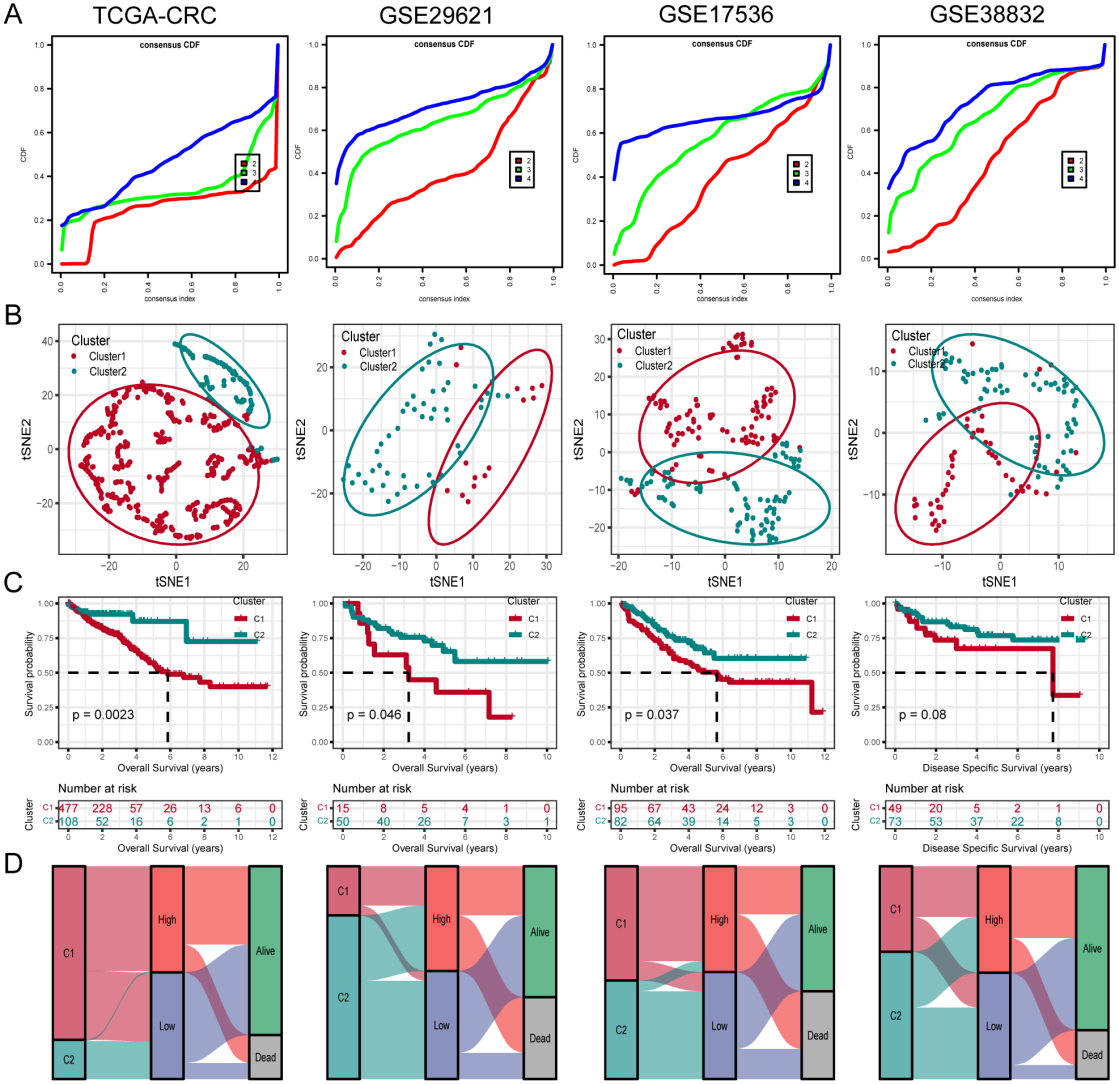
Unsupervised clustering of PCD related model genes. **(A)** Empirical cumulative distribution function plot displaying consensus distributions for each k value (from 2 to 4). **(B)** t-SNE analysis plot based on the molecular clusters. **(C)** Kaplan-Meier analysis of the prognostic differences between two molecular clusters. **(D)** Diagram showing the interrelationship between molecular clusters, survival status, and risk score groups.

To further investigate unidentified CRC subtypes, we performed CC analysis on three PCD-related model genes. The results showed that the differences between the subgroups were most pronounced at maxK = 2, suggesting that the patients with CRC could be effectively categorized into two groups (**Figure 5A**). Furthermore, t-SNE clustering analysis was conducted on the two subtypes, yielding satisfactory differentiation results (**Figure 5B**). Notably, there were significant differences in OS between the subtypes, with cluster 2 displaying a more favorable prognosis and cluster 1 showing a poorer prognosis (**Figure 5C**). Similar results were observed in the validation cohorts GSE29621, GSE17536 and GSE38832. Furthermore, analysis indicated that the majority of Cluster1 was linked to a high-risk score, while the majority of Cluster2 was correlated with a low-risk score (**Figure 5D**).

### 3.6 Establishment and assessment of the nomogram survival model

**Figure 6:**
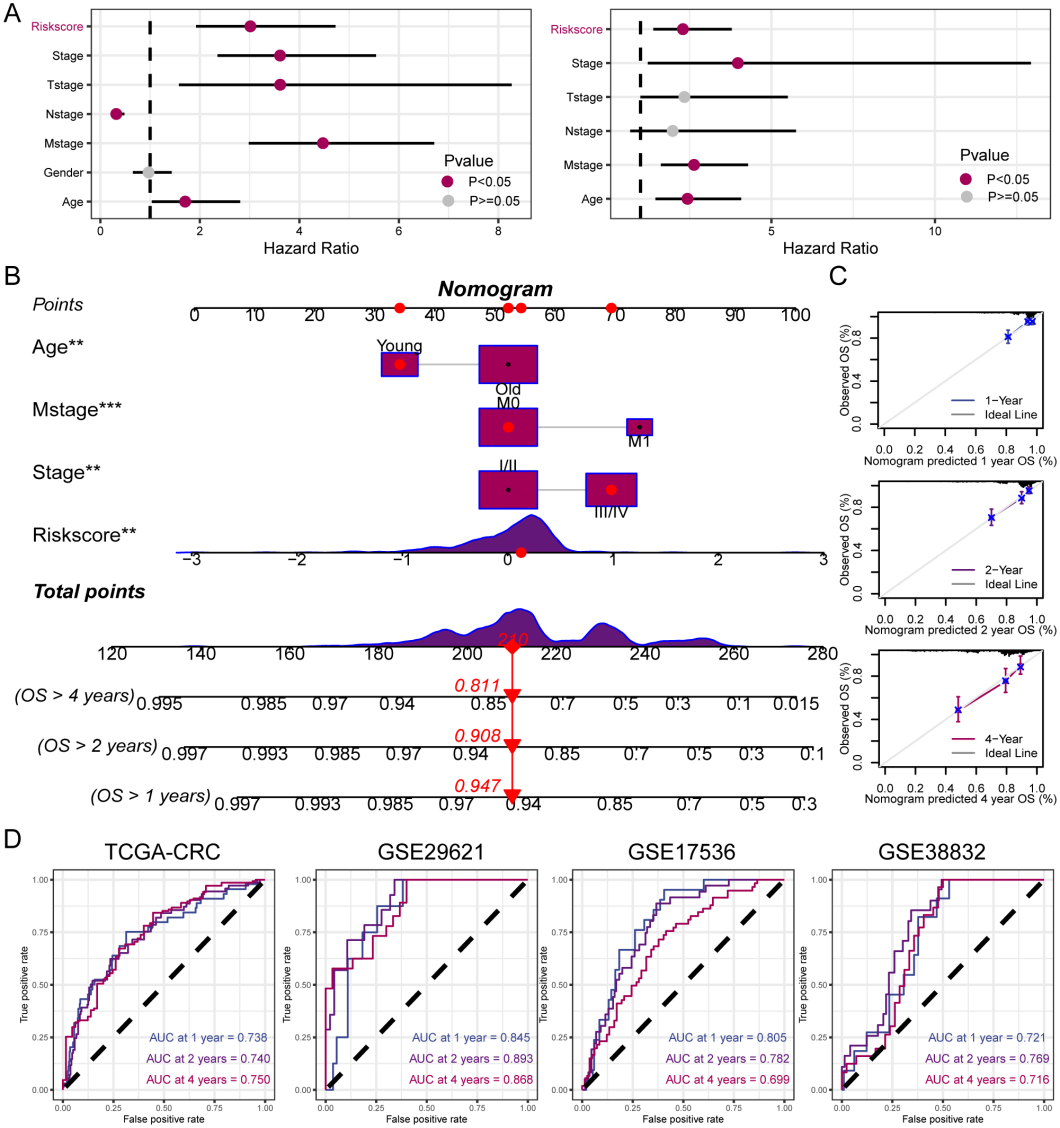
Establishment and assessment of the nomogram. **(A)** Univariate and multivariate analysis for the clinicopathologic characteristics and risk score. **(B)** A nomogram capable of predicting the prognosis of patients with CRC. **(C)** Calibration plots showing the probability of 1-, 2-, and 4-year OS. **(D)** ROC curves analysis of nomogram. **P < 0.01; ***P < 0.001.

Univariate and multivariate Cox regression analyses were performed on the clinical information and corresponding risk scores of the 585 patients from TCGA. Univariate Cox regression analysis identified that risk score, stage, T-stage, N-stage, M-stage, and age significantly affected survival. Subsequently, when these survival-influencing factors were incorporated into the Multivariate Cox regression analysis, the established risk score was determined to be an independent prognostic indicator (**Figure 6A**). A nomogram was developed for TCGA cohort utilizing Multivariate Cox regression analysis to evaluate the OS of patients with CRC at 1, 2, and 4 years. The model incorporated risk score, stage, M-stage, and age (**Figure 6B**). The calibration curve illustrates the precision of the model in predicting survival rates at these time points (**Figure 6C**). Additionally, the area under the curve values was assessed in four independent cohorts, indicating that the nomogram exhibited high accuracy in predicting survival rates at 1, 2, and 4 years for patients with CRC (**Figure 6D**).

### 3.7 Dissection of TME based on PCD-related gene signature

**Figure 7:**
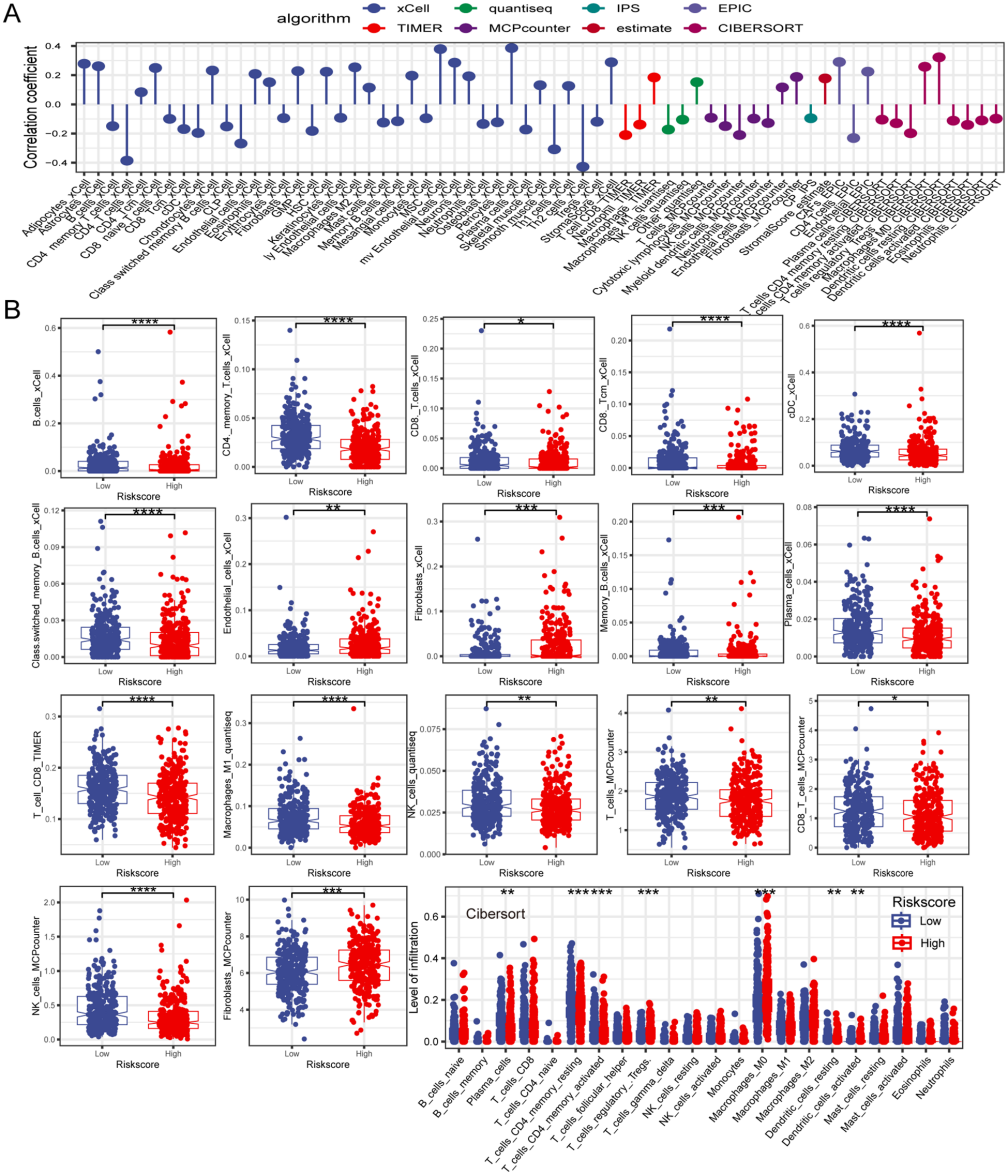
The investigation of the immune landscape in low- and high-risk groups. **(A)** Plot illustrating the correlation between risk score and levels of immune cell infiltration. **(B)** The difference in immune cell infiltration level between low- and high-risk groups. *P < 0.05; **P < 0.01; ***P < 0.001; ****P < 0.0001.

**Figure 8:**
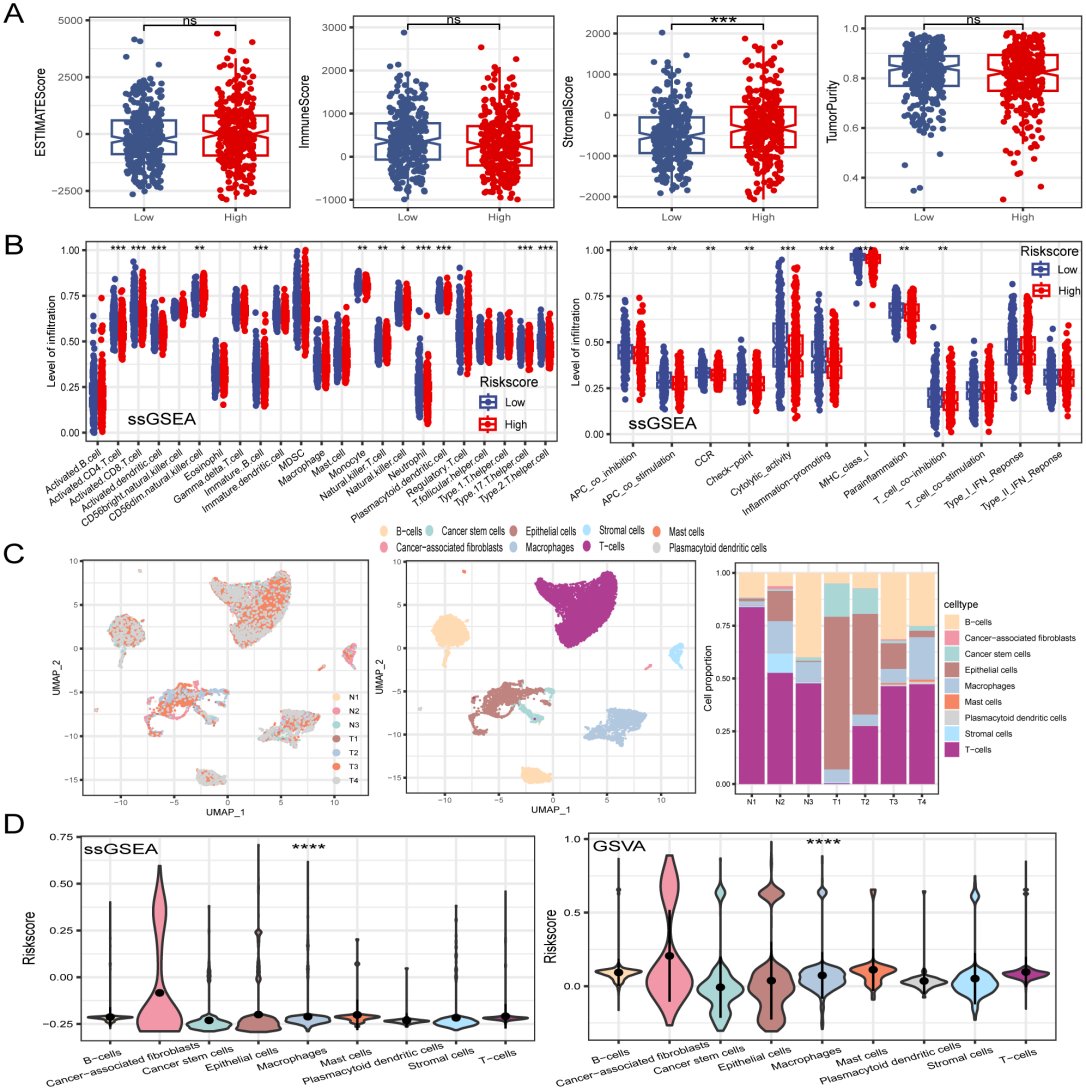
Dissection of TME based on PCD signature. **(A)** The difference of TME score between in low- and high-risk groups. **(B)** ssGSEA algorithm calculated the differences in immune cell infiltration levels and immune function between low- and high-risk groups. **(C)** Visualization of all cell subtypes from 7 samples through t-SNE plot. Different cell subtypes were annotated. Proportions of different cell types in each sample were computed. **(D)** Violin plot of risk score in different cell types. *P < 0.05; **P < 0.01; ***P < 0.001; ****P < 0.0001.

Tumor immune cell infiltration is a crucial feature of the TME. Here, we employed seven algorithms, including XCELL, TIMER, QUANTISEQ, MCPCOUNTER, ESTIMATE, EPIC, and CIBERSORT, to assess the immune cell infiltration scores of 585 patients from TCGA database. Spearman’s correlation analysis demonstrated a potential relationship between lower risk scores and heightened immune activity. The risk score inversely correlated with the infiltration of various immune cells, including B cells, CD4+ memory T cells, CD4+ T cells, CD8+ naïve T cells, CD8+ Tcm cells, cDC, and other immune cells (**Figure 7A**). This trend remained consistent across multiple algorithms, confirming the robustness and reliability of our risk score assessment approach. Additionally, the risk score showed a positive correlation with the stromal score, fibroblasts, and endothelial cells, and a negative correlation with M1 macrophages. To provide further insight, we compared the outputs from the different algorithms and noted minimal discrepancies, supporting the consistency of the observed associations. Visualization of the TME between low- and high-risk groups revealed that immune cells associated with a favorable tumor prognosis, such as B cells, CD4+ T cells, CD8+ T cells, plasma cells, M1 macrophages, and natural killer (NK) cells, had lower infiltration in the high-risk group than in the low-risk group. Conversely, elements linked to an unfavorable tumor prognosis, such as fibroblasts and endothelial cells, tended to have higher infiltration levels in the high-risk group (**Figure 7B**). These findings emphasize the differential immune landscape between risk groups, as consistently identified across algorithms. To validate the accuracy of immune infiltration and investigate the differences in immune function between the high- and low-risk groups, ssGSEA was performed. Our findings were consistent with those of previous algorithms, showing that the low-risk group had superior immune infiltration compared with the high-risk group. Moreover, the immune function scores of the low-risk group generally surpassed those of the high-risk group. Analysis using the ESTIMATE algorithm revealed that the high-risk group had remarkably higher stromal scores, but lower immune scores than the low-risk group (**Figures 8A, B**). To further explore the risk score expression in different cell types, single-cell RNA transcriptome data from GSE161277 were analyzed. Cell type annotation was conducted (**Figure 8C**), and the risk score expression in each cell was calculated using the ssGSEA and GSVA algorithms for accuracy. Violin plots consistently demonstrated that the risk score was expressed across various cell types, with tumor-associated fibroblasts exhibiting the highest expression levels. This pronounced expression in fibroblasts underscores their potential role in mediating the adverse prognostic impact associated with elevated risk scores (**Figure 8D**).

### 3.8 PCD-related gene signature was associated with immunotherapy responses in CRC

**Figure 9:**
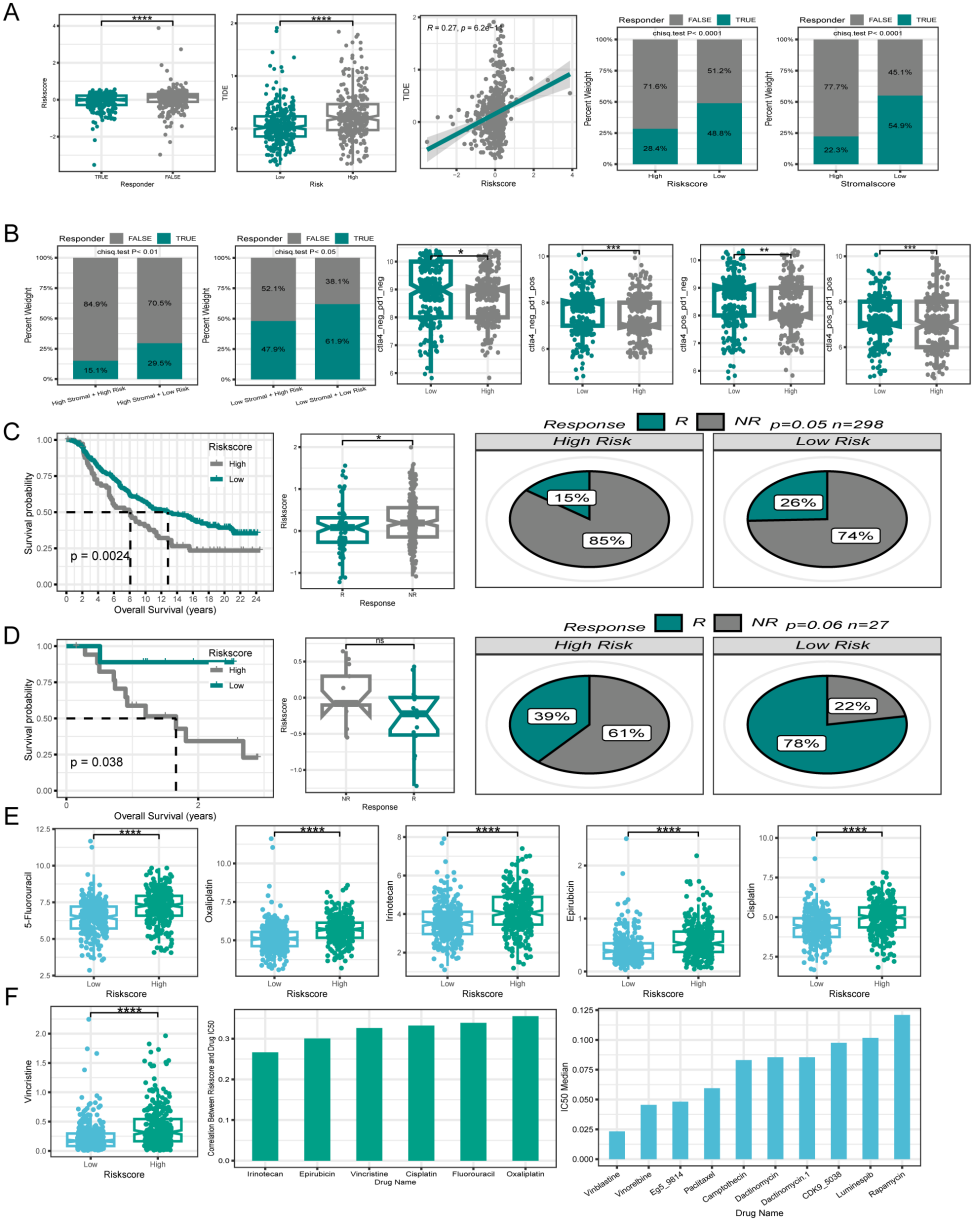
Efficacy of PCD signature in predicting therapeutic sensitivity. **(A)** Differences in risk score among patients with different responses to immunotherapy. The correlation between risk score and TIDE. Variations in immunotherapy response rates between high- and low-risk groups. Differences in immunotherapy response rates between high- and low stromal score groups. **(B)** Utilizing risk score and stromal score together to predict immunotherapy response rates. Disparities in IPS between patients in different risk groups. **(C, D)** Validation of the predictive efficiency of the PCD signature in the IMvigor210 and GSE78220 cohort. **(E)** Variations in IC50 of first-line clinical treatment drugs for CRC between high- and low-risk groups. **(F)** The correlation between riskscore and IC50 values for first-line clinical treatment drugs in CRC. The ten drugs with the lowest IC50 values selected from a pool of 198 drugs. *P < 0.05; **P < 0.01; ***P < 0.001; ****P < 0.0001.

Immunotherapy has emerged as a powerful clinical approach for treating various cancers (49). However, patients who fail to mount an effective immune response do not derive significant benefit from immunotherapies. Therefore, there is an urgent need to distinguish between immunotherapy-sensitive and immunotherapy-insensitive patients with CRC. The TIDE algorithm, which assessed the predictive power of the risk score in determining immunotherapy response in patients with CRC, revealed that responders had lower risk scores than non-responders. Notably, the low-risk group displayed lower TIDE scores and correlation analyses, indicating a positive relationship between the risk and TIDE scores. Furthermore, the low-risk group showed a higher immunotherapy response rate (48.8%) in contrast to the poorer response rate observed in the high-risk group (28.4%). Moreover, the risk score was associated with the tumor stroma, prompting an investigation into the impact of stromal scores on immunotherapy response rates. Interestingly, the high stromal score group exhibited a lower immunotherapy response rate (22.3%) compared to those in the low stromal score group. (54.9%) (**Figure 9A**). By integrating the risk and stromal scores, the findings revealed that in the low-stromal score group, the immunotherapy response rates were 61.7% and 46% in the low-risk and high-risk groups, respectively. Conversely, in the high stromal score group, the immunotherapy response rate was 32% in the low-risk group and 15% in the high-risk group. These findings suggest that the combination of the risk and stromal score could serve as a reliable biomarker for predicting immunotherapy response in patients with CRC.

Additionally, the IPS is a molecular indicator used to assess the immune status of tumors in patients, with a higher IPS suggesting a more favorable response to immunotherapy. Analysis of IPS data from the TCGA database revealed that the low-risk group generally exhibited higher IPS levels (**Figure 9B**). The relationship between the risk score and immunotherapy response rates was further validated using two independent datasets, GSE78220 and Imvigor210. Both datasets showed that the patients who responded positively to immunotherapy tended to have lower risk scores. Additionally, individuals in the low-risk category displayed higher rates of immunotherapy response. Moreover, patients with a low-risk score demonstrated similarly superior OS compared with those with a high-risk score within the immunotherapy cohort (**Figures 9C, D**).

### 3.9 Efficacy of PCD-related gene signature in predicting drug sensitivity

To investigate the relationship between PCD-related gene signatures and drug sensitivity in patients with CRC, our analysis focused on comparing the IC50 values of first-line drugs (5-Fluorouracil, Oxaliplatin, Irinotecan, Epirubicin, Cisplatin, and Vincristine) between the high- and low-risk groups of clinically treated patients with CRC. Interestingly, we observed that patients in the low-risk group demonstrated a significantly higher sensitivity to these first-line drugs (**Figure 9E**). A correlation plot revealed a positive correlation between the IC50 values of first-line drugs and the risk score, implying that patients with elevated risk score expression could potentially develop resistance to first-line drugs.

To explore new therapeutic possibilities for patients insensitive to first-line CRC treatment drugs, we calculated the median IC50 values of 198 chemotherapy/targeted drugs. The top ten drugs with the lowest IC50 values (Vinblastine, Vinorelbine, Eg5, Paclitaxel, and Camptothecin, etc.) were identified in ascending order. The very low IC50 values suggest the potential sensitivity of patients with CRC to these drugs, making them potential candidates for those who are insensitive to first-line treatment drugs (**Figure 9F**).

### 3.10 Biological function of the selected gene

**Figure 10:**
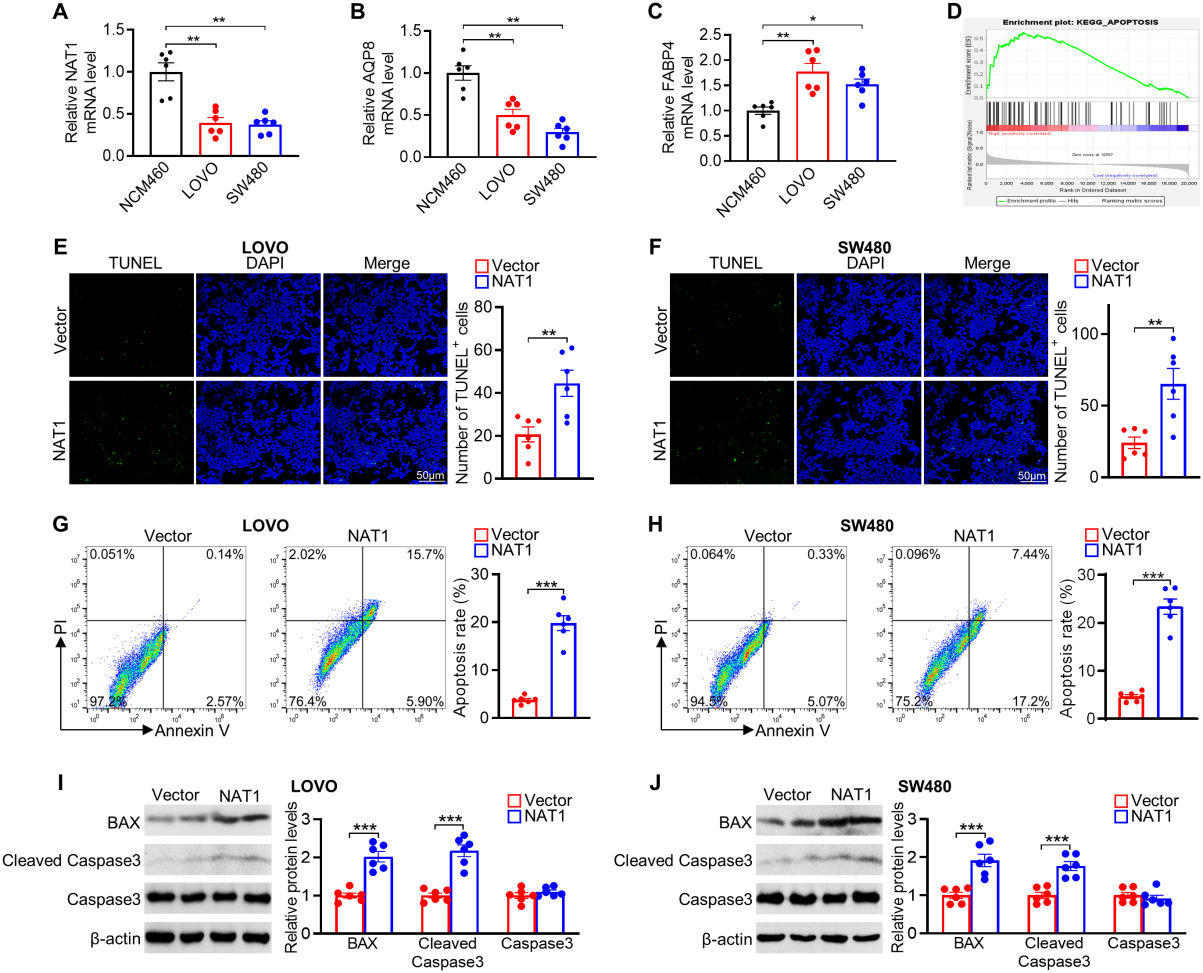
Experimental verification of 3 genes expression and role of NAT1 in mediating apoptosis in colorectal cancer cells. **(A–C)** The mRNA levels of NAT1 **(A)**, AQP8 **(B)**, and FABP4 **(C)** in NCM460 normal colonic and LOVO and SW480 colorectal cancer cells. **(D)** GSEA of apoptosis pathway in the NAT1-high subgroups. **(E, F)** Representative images of the TUNEL assays exposed to NAT1 overexpression in LOVO **(E)** and SW480 **(F)** cells, respectively. **(G, H)** Apoptosis was evaluated by Annexin V/PI staining with a flow cytometer. **(I, J)** Western blot results for analysis of BAX and cleaved caspase-3 expression treated with NAT1 overexpression. n=6 for each group. *P < 0.05; **P < 0.01; ***P < 0.001.

To verify the expression of model genes, qPCR showed that NAT1 and AQP8 had significantly lower expression levels, while FABP4 showed higher expression in CRC cell lines (LOVO and SW480) compared with that in NCM460 normal cells (**Figures 10A–C**). Interestingly, the results of FABP4 mRNA levels seemed to be inconsistent with the analysis from the TCGA database. This discrepancy may be due to differences in the cell lines and tumor tissues used to measure mRNA expression. We focused on exploring the role of NAT1 in CRC since NAT1 yielded the largest contribution to the risk model. GSEA revealed that apoptosis signaling pathway genes were enriched in the high NAT1 expression phenotype (**Figure 10D**). Strikingly, NAT1 overexpression was correlated with increased tumor cell apoptosis, as determined by TUNEL and Annexin V/PI staining (**Figures 10E–H**). Consistent with these results, NAT1 overexpression increased the expression of apoptosis-related proteins, including BAX and cleaved caspase-3 (**Figures 10I, J**). Collectively, these findings suggested that NAT1 dramatically activated apoptosis in CRC cells.

## 4 Discussion

This study provides a comprehensive analysis of 14 distinct PCD types, resulting in the identification of crucial PCD-related genes in CRC and the development of a novel risk score. The outstanding predictive performance of the risk score was extensively validated using multiple external cohorts. Furthermore, we integrated the risk score with patients’ clinical characteristics to construct a nomogram, enhancing its clinical relevance and further strengthening its predictive accuracy. Our findings reveal a significant association between the risk score and the TME, immunotherapy responsiveness, and drug sensitivity, highlighting its clinical applicability in precision oncology. Existing CRC prognostic models typically focus on single-gene markers or individual PCD pathways, such as PD-L1, ferroptosis, or cuproptosis alone (50–52). In contrast, our model uniquely integrates multiple PCD pathways, enabling a comprehensive analysis to identify the genes that play a more pivotal role in prognosis, which are subsequently used to construct a robust predictive model. This multi-dimensional approach not only enhances the accuracy of patient stratification but also provides a more nuanced prediction of therapeutic outcomes, encompassing both immunotherapy and chemotherapy responses. The robustness of our model is further underscored by its validation through single-cell transcriptomic data, reflecting its adaptability and reliability. By capturing the complexity of the TME and the diverse PCD pathways involved, our model offers an innovative and personalized framework for optimizing CRC patient management, positioning it as a superior prognostic tool compared to existing models.

Increasing evidence suggests that PCD plays a fundamental role in biological processes and has long been linked to the development and metastasis of cancer (44). Our established signature, composed of three genes associated with PCD (NAT1, AQP8, and FABP4), has been shown to accurately predicted OS and DSS in patients with CRC. Each of these model genes fulfills unique functions in CRC progression via distinct mechanisms. FABP4, a low-molecular-weight protein responsible for transporting long-chain fatty acids and other hydrophobic ligands, has a significant impact on tumor initiation, progression, and treatment (53). In ovarian cancer cells, enhanced expression of FABP4 induced by adipocytes not only promotes metastasis but also mediates resistance to carboplatin (54). In pancreatic cancer, FABP4 facilitates the movement, invasion, and spread of cancer cells by influencing signals related to epithelial-mesenchymal transition (EMT) (55). Consistent with previous studies, Kaplan-Meier analysis demonstrated that patients with CRC with high FABP4 expression exhibited a lower survival rate, suggesting that FABP4 is a potential risk factor for CRC. AQP8 functions as a water-selective transporter (56). Our analysis indicated that patients with CRC with low AQP8 expression had a poorer prognosis. Previous studies have shown that AQP8 overexpression can lead to a notable decrease in the growth, invasiveness, and colony formation of SW480 and HT-29 CRC cells. This is possibly due to AQP8 overexpression, which inhibits the PI3K/AKT signaling pathway and PCDH7 expression, ultimately suppressing oncogenic characteristics (57). NAT1 is a phase II xenobiotic-metabolizing enzyme that is widely distributed in various tissues (58). Our results indicate that elevated levels of NAT1 expression are correlated with better outcomes in patients with CRC. Furthermore, NAT1 emerged as a key gene in our model, with the highest contribution from the risk model. Therefore, we focused on this gene in the present study. Initial validation of NAT1 expression, supported by qPCR results aligned with TCGA-CRC data, indicated reduced expression in tumors. Furthermore, our exploration of the relationship between NAT1 expression and PCD through GSEA demonstrated the upregulation of the apoptotic pathway in response to high NAT1 expression. Interestingly, previous studies show that NAT1 knockdown promotes apoptosis in HT-29 cells under low glucose conditions (59). This seems contrary to our results, which revealed that NAT overexpression led to apoptosis in two different cell lines (LOVO and SW480). A possible explanation is that the regulatory role of NAT1 in apoptosis varies across HT-29, LoVo, and SW480 cells, depending on the p53 status and cellular context. In HT-29 cells, NAT1 stabilizes the gain-of-function mutant p53 (R273H) under low-glucose conditions, thereby modulating its activity. This stabilization enables the mutant p53 to effectively reduce ROS accumulation, which, in turn, suppresses glucose deprivation-induced apoptosis (59). In contrast, in LoVo cells, where p53 is wild-type and retains its pro-apoptotic function under cellular stress (60), NAT1 overexpression may increase ROS levels or alter metabolic pathways, further activating p53’s apoptotic response, leading to increased cell death. Similarly, in SW480 cells, despite the presence of mutant p53 (R273H and P309S) (61), the mutation type differs from that in HT-29 cells, potentially preventing NAT1 from exerting the same stabilizing effect, and instead, inducing apoptosis through alternative mechanisms, such as ROS elevation. This indicates that the function of NAT1 varies significantly depending on the p53 background and cellular stress conditions, highlighting the importance of investigating NAT1’s role in specific cell types and contexts.

Disrupted microenvironments significantly influence the occurrence and development of tumors. The TME aids tumors in evading immune surveillance and drug interference. The three model genes play distinct roles within the TME. FABP4 regulates lipid metabolism, enhancing fatty acid transport and activating pro-tumorigenic pathways, which promote the accumulation of tumor-associated macrophages (TAMs) and myeloid-derived suppressor cells (MDSCs), leading to immune suppression and tumor immune evasion (62). AQP8 modulates ROS levels within the TME, influencing immune cell behavior and promoting the recruitment of suppressive cells like Regulatory T cells (Tregs) and MDSCs, which create an immunosuppressive environment that supports tumor progression (63). NAT1 modulates the TME by regulating immune cell infiltration levels, particularly affecting Cytotoxic T Lymphocytes (CTLs), NK cells, and TAMs. Downregulation of NAT1 correlates with decreased CTL and NK cell infiltration, promoting an immunosuppressive environment characterized by increased TAM and Treg presence, which collectively facilitates tumor progression and metastasis (64–66). In the TME, various cell types collectively contribute to tumor development, significantly impacting patient prognosis (67). M1 macrophages, known for their “classical activation,” produce type I pro-inflammatory cytokines, engage in antigen presentation, and possess anti-tumorigenic properties (68). However, cancer-associated fibroblasts contribute to pro-tumorigenic functions (69). The tumor vasculature sustained by the TME supports continuous tumor growth (70). Endothelial cells contribute to the formation of new blood vessels by regulating angiogenesis (71). The initiation of primary tumor invasion involves the critical process of EMT, where tumor cells undergo a transformation marked by the loss of epithelial markers and the acquisition of mesenchymal traits. This phenotypic shift confers stem cell-like properties and a migratory phenotype to tumor cells, supported by the altered immune microenvironment (72). Several studies have shown that the stroma plays an essential role in cancer phenotype transformation (73). CD8+ T cells significantly correlated with recurrence time, DFS, and OS in patients (74). Our analysis aligns with the findings of previous research. Patients with high-risk scores and poor prognoses displayed decreased levels of beneficial immune cells, such as CD8+ T cells, CD4+ T cells, M1 macrophages, and NK cells, whereas cells that support tumor progression, such as endothelial cells, tumor-associated fibroblasts, and stromal infiltration, were elevated. These patterns were consistent across various algorithms, elucidating the basis of unfavorable prognosis in this patient cohort. As the risk score increases, immune cell infiltration decreases, tumor stromal components increase, and immune function deteriorates, creating an imbalanced TME that facilitates tumor formation and invasion. Consequently, patients with high-risk scores had lower survival rates and poorer prognoses.

Immunotherapy has revolutionized the treatment of patients with unresectable cancers (49). Biomarkers, such as PD-1 and PD-L1 are valuable for predicting the effectiveness of immunotherapy. However, the interplay between these biomarkers is intricate, and it is unclear whether combining them enhances the efficacy compared to using a single marker (75). This study investigated the correlation between the risk score and effective immunotherapy biomarkers. Results from the ssGSEA immune checkpoint analysis showed that the low-risk group exhibited superior performance. Furthermore, the application of the TIDE score, which indicates immune escape likelihood and ICI treatment effectiveness, showed a significantly higher TIDE score in the high-risk group. The positive correlation between the risk score and TIDE score suggested that patients with lower risk scores derived greater benefits from ICI therapy. By combining the stromal and risk scores, we were able to predict the responsiveness of patients with CRC to immunotherapy more accurately. The IPS, developed from the TCGA RNA-seq data to predict patient responses to immune checkpoint inhibitors (ICIs) (76), also indicated a better response in the low-risk group. Taking all these immunotherapy-related findings into consideration, it appears that patients in the low-risk group may benefit more from immunotherapy in the context of CRC treatment.

Pharmacological interventions, encompassing chemotherapy and targeted therapies, are pivotal in enhancing survival outcomes for patients with CRC (77). Our results indicated a correlation between the risk score and IC50 values of chemotherapy/targeted drugs in patients with CRC. The IC50 values were calculated for 198 chemotherapy/targeted drugs, revealing that patients in the low-risk group exhibited higher sensitivity to the first-line clinical treatment drugs for CRC than those in the high-risk group. Furthermore, correlation analysis revealed a positive relationship between the risk score and the IC50 values of first-line clinical treatment drugs, indicating that as the risk score increases, patients with CRC may develop resistance to these drugs. To identify potential alternative treatments for patients with CRC, we arranged the median IC50 values of non-first-line drugs in ascending order and pinpointed the ten drugs with the lowest values. These drugs are promising for the treatment of patients unresponsive to first-line drugs. Consequently, it is plausible to consider that patients at high-risk may derive lesser benefits from first-line drug treatments and might require alternative drugs or treatment strategies.

The PCD-related gene signature developed in this study offers a practical and translational tool for CRC management, enabling precise patient stratification through risk score calculation. In clinical settings, gene expression levels of FABP4, AQP8, and NAT1 can be measured from routine biopsy samples using established techniques such as qPCR or RNA sequencing. These technologies, widely available in modern hospitals, allow for the rapid and accurate quantification of gene expression, integrating seamlessly into clinical workflows. This stratification guides personalized treatment decisions, such as prioritizing low-risk patients for standard chemotherapy or identifying high-risk individuals for alternative or immunotherapy approaches, thereby maximizing therapeutic efficacy and improving clinical outcomes. Thus, the integration of gene expression analysis and risk score computation in hospitals is not only feasible but also streamlined for clinical application.

While our risk model demonstrates strong performance across TCGA-CRC and external cohorts, its generalizability may be limited due to the demographic and geographic diversity of the sample populations, potentially restricting its applicability to broader CRC populations. Computational methods, including XCELL, TIMER, and others, were employed to analyze the TME and immune cell infiltration, yet these algorithms may not fully capture the dynamic and complex immune interactions. Although IC50 values provide preliminary insights into drug sensitivity, further experimental and clinical studies are needed to validate their efficacy and safety. Future research should incorporate multi-ethnic cohorts and utilize single-cell RNA sequencing and spatial transcriptomics to better characterize TME heterogeneity and validate the mechanistic roles of hub genes (e.g., NAT1, AQP8, FABP4).

In summary, our study highlights the unique advantages of utilizing multigene combinations over single genes for predicting cancer prognosis. The risk model, which was derived from hub genes across 14 types of PCD, plays a crucial role in the diagnosis and prognosis of patients with CRC. Furthermore, our study offers a straightforward approach for categorizing patients into high- or low-risk subgroups. Finally, we conducted separate investigations on TME variances, mutation statuses, immune therapy responses, chemotherapy drug sensitivities, and other factors in these subgroups. This study offers valuable insights into the tailoring of personalized treatment plans for patients with CRC.

## 5 Conclusions

We developed a risk model for patients with CRC. This model showed a significant correlation between the risk score and various factors such as patient prognosis, TME, response to immunotherapy, and sensitivity to chemotherapy drugs. Patients classified in the low-risk group demonstrated better prognoses and higher response rates to immunotherapy and first-line chemotherapy drugs compared to those in the high-risk group. Conversely, patients in the high-risk group may experience resistance to first-line CRC chemotherapy drugs as their risk score increases. Through the use of regression methods, 10 drugs were identified as sensitive for CRC treatment, offering potential for patients who do not respond well to standard first-line medications. To further elucidate the molecular mechanisms underlying the model genes, NAT1, as the most pivotal gene within the risk model, was selected for subsequent fundamental experimental investigations. The findings suggested that NAT1 may play a role in CRC progression by impacting cell apoptosis.

## Data Availability

The datasets presented in this study can be found in online repositories. The names of the repository/repositories and accession number(s) can be found in the article/**Supplementary Material**.

## Supplementary material

Supplementary Figure S1 relationship between expression levels of the three model genes and OS separately.

Supplementary Table S1 programmed cell death gene.

Supplementary Table S2 enrichment analysis of high- and low- risk groups; Data; R script; AQP8、FABP4、NAT1_QPCR; full_membranes_WB.
