# Supplementary material for: Identification of key programmed cell death genes for predicting prognosis and treatment sensitivity in colorectal cancer
Source: Front Oncol. 2024 Nov 13;14:1483987. doi: 10.3389/fonc.2024.1483987 (PMC11603958; doi:10.3389/fonc.2024.1483987)
Supplement: Supplementary Figure S1 — relationship between expression levels of the three model genes and OS separately. [file DataSheet1.zip › Figure S1 & R script.docx]

Supplementary Material

# Supplementary Data

## R script

Model construction :

rm(list = ls())

library(tidyverse)

GENE1 <- read.delim("mode_gene.txt")%>%as.matrix

exp <- data.table::fread("TCGA-CRC_tpm_mRNA_clini.txt",data.table = F)

exp <- exp %>% column_to_rownames("patient_ID") %>% t() %>% as.data.frame()

exp <- exp[GENE1,] %>% na.omit()

table(is.na(exp))

dat1 <- data.table::fread("TCGA-CRC_Clinical.txt",data.table = F)

rownames(dat1) <- dat1$patient_ID

rt <- dat1 %>% dplyr::select(futime="time",fustat="status")

#rt <- rt %>% filter(futime > 1/12)

exp1 <- exp %>% t() %>% as.data.frame()

aa <- intersect(rownames(rt),rownames(exp1))

rt <- rt[aa,]

exp1 <- exp1[aa,]

exp2 <- scale(exp1)

identical(rownames(rt),rownames(exp2))

surv.expr <- cbind(rt,exp2)

#write.table(surv.expr, file='surv_expr_TCGA.txt',sep = "\t",row.names = T,col.names = NA,quote = F)

library(survival)

Coxoutput <- NULL

for(i in 3:ncol(surv.expr)){

g <- colnames(surv.expr)[i]

cox <- coxph(Surv(futime,fustat) ~ surv.expr[,i], data = surv.expr)

coxSummary = summary(cox)

Coxoutput <- rbind.data.frame(Coxoutput,

data.frame(gene = g,

HR = as.numeric(coxSummary$coefficients[,"exp(coef)"])[1],

z = as.numeric(coxSummary$coefficients[,"z"])[1],

pvalue = as.numeric(coxSummary$coefficients[,"Pr(>|z|)"])[1],

lower = as.numeric(coxSummary$conf.int[,3][1]),

upper = as.numeric(coxSummary$conf.int[,4][1]),

stringsAsFactors = F),

stringsAsFactors = F)

}

#write.table(Coxoutput, file = "COX_TCGA.txt",sep = "\t",row.names = F,col.names = T,quote = F)

Coxoutput1 <- Coxoutput[which(Coxoutput$pvalue < 0.05),]

hr <- round(Coxoutput1$HR,3)

CI_LL <- round(Coxoutput1$lower,3)

CI_HL <- round(Coxoutput1$upper,3)

HR <- paste0(hr,"(", CI_LL, "-", CI_HL, ")")

p <- round(Coxoutput1$pvalue,6)

xt_output1 <- data.frame(Gene = Coxoutput1$gene,

HR_95CI = HR,

pvalue = p)

#write.csv(xt_output1,file = "xt_output1.csv",row.names = F)

gene1 <- Coxoutput1$gene

rt1 <- surv.expr[,c(1,2)]

rt2 <- surv.expr[,gene1]

rt <- cbind(rt1,rt2)

library(glmnet)

library(survival)

str(rt)

set.seed(1)

x=as.matrix(rt[,c(3:ncol(rt))])

y=data.matrix(Surv(rt$futime,rt$fustat))

fit=glmnet(x, y, family = "cox", maxit = 100000,alpha = 1)

pdf(file="lasso1.pdf", height= 6.5, width= 6.5)

plot(fit, xvar = "lambda", label = FALSE)

cvfit = cv.glmnet(x, y, family="cox", maxit = 100000,alpha = 1)

dev.off()

pdf(file="lasso2.pdf", height= 5, width= 5)

plot(cvfit)

abline(v = log(c(cvfit$lambda.min,cvfit$lambda.1se)),lty="dashed")

dev.off()

library(tidyverse)

coef=coef(fit, s = cvfit$lambda.min)

index=which(coef != 0)

actCoef=coef[index]

lassoGene=row.names(coef)[index]

geneCoef=cbind(Gene=lassoGene,Coef=actCoef)

geneCoef

lassoGene1=row.names(coef)[index]

lassoGene1=c("futime","fustat",lassoGene)

coef_table <- geneCoef %>% as.data.frame()

coef_table$coeff <- round(as.numeric(coef_table$Coef),5)

#write.table(coef_table, file='coef_table_lasso1.txt',sep = "\t",row.names = T,col.names = NA,quote = F)

aa <- lassoGene

rt1 <- rt[,c(1,2)]

train <- rt[,aa]

train <- cbind(rt1,train)

multiCox <- coxph(Surv(futime, fustat) ~ ., data = train)

multiCox=step(multiCox,direction = "both")

multiCoxSum=summary(multiCox)

outMultiTab=data.frame()

outMultiTab=cbind(

coef=round(multiCoxSum$coefficients[,"coef"],3),

HR=multiCoxSum$conf.int[,"exp(coef)"],

lower=multiCoxSum$conf.int[,"lower .95"],

upper=multiCoxSum$conf.int[,"upper .95"],

pvalue=multiCoxSum$coefficients[,"Pr(>|z|)"])

outMultiTab=cbind(id=row.names(outMultiTab),outMultiTab) %>% as.data.frame()

outMultiTab <- outMultiTab[which(outMultiTab$pvalue < 0.05),]

str(outMultiTab)

#write.table(outMultiTab, file='TCGA_muticox.txt',sep = "\t",row.names = T,col.names = NA,quote = F)

actCoef <- round(as.numeric(outMultiTab$coef),3)

lsg <- rownames(outMultiTab)

FinalGeneExp = rt[,lsg]

myFun = function(x){crossprod(as.numeric(x),actCoef)}

riskScore = apply(FinalGeneExp,1,myFun)

outCol = c(lsg)

risk = as.vector(ifelse(riskScore > median(riskScore), "high", "low"))

trainRiskOut = cbind(rt[,c("futime","fustat",outCol)], riskScore=as.vector(riskScore), risk)

coef_table1 <- outMultiTab %>% as.data.frame()

colnames(coef_table1)[1:2] <- c("Gene","Coef")

#write.table(coef_table1, file='coef_table.txt',sep = "\t",row.names = T,col.names = NA,quote = F)

#write.table(trainRiskOut, file='TCGA_group.txt',sep = "\t",row.names = T,col.names = NA,quote = F)

diff=survdiff(Surv(futime, fustat) ~risk,data = train)

pValue=1-pchisq(diff$chisq,df=1)

library(timeROC)

library(survival)

library(survminer)

fit <- survfit(Surv(futime, fustat)~ risk, data =trainRiskOut )

summary(fit)

pround <- round(pValue,9)

pdf("TCGA-CRC_KM.pdf",wi=5,he=5)

ggsurvplot(fit,

data = trainRiskOut,

palette =c("#EE0000FF","#3B4992FF"),

pval = pround,

risk.table = T)

dev.off()

library(pheatmap)

library(tidyverse)

rt=trainRiskOut[order(trainRiskOut$riskScore),]

str(rt)

riskClass <- rt[,"risk"]

lowLength <- length(riskClass[riskClass == "low"])

highLength <- length(riskClass[riskClass == "high"])

point <- rt[,"riskScore"]

point[point>10]=10

range(point)

pdf("1.pdf",wi=3.5,he=3.5)

plot(point,

type="p",

pch=20,

xlab="Patients (increasing risk socre)",

ylab="Risk score",

col=c(rep("#3B4992FF",lowLength),

rep("#EE0000FF",highLength)))

abline(h=median(rt$riskScore), v=lowLength,lty=2)

legend("topleft",

c("High risk", "Low risk"),

bty="n",

pch=19,

col=c("#EE0000FF","#3B4992FF"),cex=1)

dev.off()

color <- as.vector(rt$fustat)

color[color==1] <- "#EE0000FF"

color[color==0] <- "#3B4992FF"

table(color)

pdf("2.pdf",wi=3.5,he=3.5)

plot(rt$futime,

pch=19,

xlab="Patients (increasing risk socre)",

ylab="Survival time (years)",

col=color,ylim=c(0,12))

abline(v=lowLength,lty=2)

legend("topleft",

c("Dead", "Alive"),

bty="n",pch=19,

col=c("#EE0000FF","#3B4992FF"),cex=1)

dev.off()

t-SNE :

rm(list = ls())

library(tidyverse)

library(Rtsne)

library(ggplot2)

data <- read.delim("TCGA_group.txt",row.names = 1)

hc_geneExp1 <- data[,3:5] %>% t() %>% as.data.frame()

group <- data %>% select(risk)

group$risk <- factor(group$risk,levels=c("low","high"))

identical(rownames(group),colnames(hc_geneExp1))

set.seed(123456)

tSNE_res <- Rtsne(t(hc_geneExp1),

dims=2,

perplexity=10,

verbose=F,

max_iter=500,

check_duplicates=F)

tsne <- data.frame(tSNE1 = tSNE_res[["Y"]][,1],

tSNE2 = tSNE_res[["Y"]][,2],

cluster = group$risk)

ggplot(tsne,

aes(x = tSNE1,

y = tSNE2,

color =cluster)) +

geom_point(size = 1)+

scale_color_manual(values= c("#3B4992FF","#EE0000FF")) +

theme_bw() +

stat_ellipse(level = 0.85, show.legend = F) +

theme(legend.position = "top")

dev.off()

Consensus Cluster :

rm(list = ls())

library(tidyverse)

exp <- data.table::fread("TCGA-CRC_tpm_mRNA_clini.txt",data.table = F)

exp <- exp %>% column_to_rownames("patient_ID") %>% scale() %>% t() %>% as.data.frame()

rt <- read.delim("TCGA_muticox.txt")

genes <- rt$Gene

hc_geneExp <- exp[genes, ]

hc_geneExp <- as.matrix(hc_geneExp)

library(ConsensusClusterPlus)

library(tidyverse)

library(survival)

library(survminer)

library(ggplot2)

library(Rtsne)

Cluster <- ConsensusClusterPlus(d = hc_geneExp,

maxK = 4,

reps = 1000,

pItem = 0.8,

clusterAlg = "km",

innerLinkage = "ward.D2",

finalLinkage = "ward.D2",

distance = "euclidean", #

seed = 66, #

plot = "pdf",

title = "Consensus Cluster")

annCol <- data.frame(Cluster = paste0("Cluster",

Cluster[[2]][["consensusClass"]]),

row.names = colnames(hc_geneExp))

cluster_group <- annCol %>% as.data.frame() %>% arrange(Cluster)

table(cluster_group$Cluster)

identical(rownames(cluster_group),colnames(hc_geneExp))

write.csv(cluster_group,"TCGA-CRC_cluster_group.csv",row.names = T)

cluster_group <- read.csv("TCGA-CRC_cluster_group.csv",row.names = 1)

hc_geneExp1 <- hc_geneExp %>% as.data.frame()

hc_geneExp1 <- hc_geneExp1[,rownames(cluster_group)]

identical(rownames(cluster_group),colnames(hc_geneExp1))

tSNE_res <- Rtsne(t(hc_geneExp1),

dims=2,

perplexity=10,

verbose=F,

max_iter=500,

check_duplicates=F)

tsne <- data.frame(tSNE1 = tSNE_res[["Y"]][,1],

tSNE2 = tSNE_res[["Y"]][,2],

cluster = cluster_group$Cluster)

set.seed(123456)

ggplot(tsne,

aes(x = tSNE1,

y = tSNE2,

color = cluster)) +

geom_point(size = 1,

alpha = 1)+

scale_color_manual(values= c("#BB0021FF","#008280FF")) +

theme_bw() +

stat_ellipse(level = 0.85, show.legend = F) +

theme(legend.position = "top")

dev.off()

dat1 <- data.table::fread("TCGA-CRC_Clinical.txt",data.table = F)

dat1 <- dat1 %>% column_to_rownames("patient_ID")

rt <- dat1 %>% select(futime="time",fustat="status")

aa <- intersect(rownames(rt),rownames(cluster_group))

rt <- rt[aa,]

cluster_group <- cluster_group[aa,,drop= F]

identical(rownames(rt),rownames(cluster_group))

rt <- cbind(rt,cluster_group)

fitd <- survdiff(Surv(futime, fustat) ~ Cluster ,

data = rt)

pValue <- 1 - pchisq(fitd$chisq, length(fitd$n) - 1)

pValue

fit <- survfit(Surv(futime, fustat)~ Cluster, data = rt)

summary(fit)

pround <- round(pValue,6)

ggsurvplot(fit,

data = rt,

palette =c("#BB0021FF","#008280FF"),

risk.table = T,

pval = pround)

dev.off()

TME :

rm(list=ls())

library(tidyverse)

library(IOBR)

data=read.delim("TCGA-CRC_tpm_mRNA_clini.txt",row.names = 1)

data=as.data.frame(t(data))

### 1.cibersort ### ------------------------------------------------------------

cibersort=deconvo_tme(eset = data, method = "cibersort", arrays = F, perm = 1000)

#write.table(cibersort,"cibersort.txt",sep = "\t",quote = F,row.names = F)

### 2.estimate ### -------------------------------------------------------------

estimate=deconvo_tme(eset = data, method = "estimate")

#write.table(estimate,"estimate.txt",sep = "\t",quote = F,row.names = F)

### 3.epic ### -----------------------------------------------------------------

epic=deconvo_tme(eset = data, method = "epic", arrays = F)

#write.table(epic,"epic.txt",sep = "\t",quote = F,row.names = F)

### 4.mcp_counter ### ----------------------------------------------------------

mcp=deconvo_tme(eset = data, method = "mcpcounter")

#write.table(mcp,"mcp.txt",sep = "\t",quote = F,row.names = F)

### 5.xcell ### ----------------------------------------------------------------

xcell=deconvo_tme(eset = data, method = "xcell", arrays = F)

#write.table(xcell,"xcell.txt",sep = "\t",quote = F,row.names = F)

### 6.timer ### ----------------------------------------------------------------

timer=deconvo_tme(eset = data, method = "timer", group_list = rep("stad",dim(data)[2]))

#write.table(timer,"timer.txt",sep = "\t",quote = F,row.names = F)

### 7.quanTIseq ### ------------------------------------------------------------

quantiseq=deconvo_tme(eset = data, tumor = TRUE, arrays = F, scale_mrna = TRUE, method = "quantiseq")

#write.table(quantiseq,"quantiseq.txt",sep = "\t",quote = F,row.names = F)

### 8.ips ### ------------------------------------------------------------------

ips=deconvo_tme(eset = data, method = "ips", plot= FALSE)

#write.table(ips,"ips.txt",sep = "\t",quote = F,row.names = F)

tme_combine<-cibersort %>%

inner_join(.,mcp,by = "ID") %>%

inner_join(.,xcell,by = "ID") %>%

inner_join(.,epic,by = "ID") %>%

inner_join(.,estimate,by = "ID") %>%

inner_join(.,timer,by = "ID") %>%

inner_join(.,quantiseq,by = "ID") %>%

inner_join(.,ips,by = "ID")

dim(tme_combine)

write.table(tme_combine,"tme_combine.txt",sep = "\t",quote = F,row.names = F)

drug sensitivity :

rm(list = ls())

library(oncoPredict)

library(data.table)

library(ggplot2)

library(ggpubr)

testExpr <- read.table("TCGA-CRC_tpm_mRNA_clini.txt",sep = "\t",row.names = 1,check.names = F,stringsAsFactors = F,header = T)

testExpr <- t(testExpr)

GDSC2_Expr <- readRDS("GDSC2_Expr (RMA Normalized and Log Transformed).rds")

GDSC2_Res <- readRDS("GDSC2_Res.rds")

GDSC2_Res <- exp(GDSC2_Res)

identical(rownames(GDSC2_Res),colnames(GDSC2_Expr))

calcPhenotype(trainingExprData = GDSC2_Expr,

trainingPtype = GDSC2_Res,

testExprData = testExpr,

batchCorrect = 'standardize',

powerTransformPhenotype = TRUE,

removeLowVaryingGenes = 0.2,

minNumSamples = 10,

printOutput = TRUE,

removeLowVaringGenesFrom = 'rawData' )

res <- fread("calcPhenotype_Output\\DrugPredictions.csv")

res <- as.data.frame(res)

rownames(res) <- res$V1

res <- res[,-1]

group1 <- read.delim("TCGA_group.txt",row.names = 1)

res <- res[rownames(group1),]

identical(rownames(group1),rownames(res))

group <- group1[,7]

for (i in 1:198) {

Cam <- as.data.frame(res[,i])

colnames(Cam) <- "senstivity"

Cam$Risk <- group

boxplot=ggboxplot(Cam, x="Risk", y="senstivity", fill="Risk",

xlab="Risk",

ylab=paste0(colnames(res)[i], " senstivity (IC50)"),

legend.title="Risk",

palette=c("#0073C2FF", "#EFC000FF")

)+

stat_compare_means(aes(label = ..p.signif..),comparisons = list(c("low","high")),method="wilcox.test")+

pdf(file=paste0(colnames(res)[i], ".pdf"), width=5, height=5)

print(boxplot)

dev.off()

}

# Supplementary Figures and Tables

## Supplementary Figures


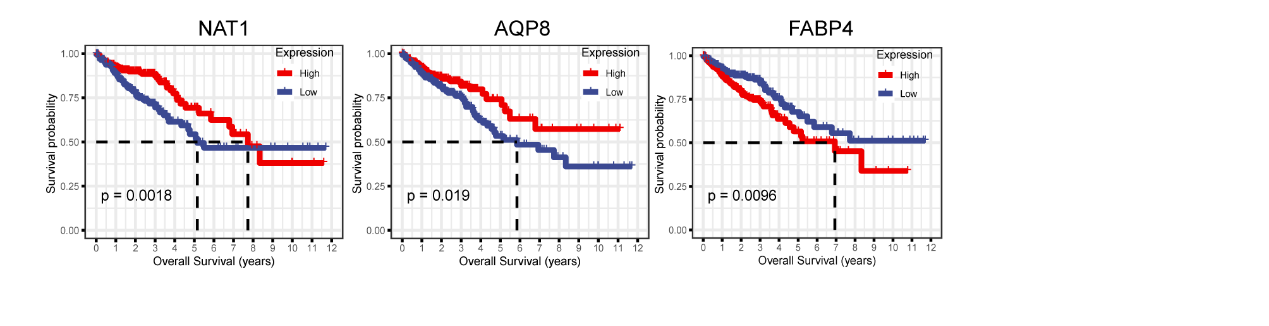


**Figure S1.** Relationship between expression levels of the three model genes and OS separately.
