# Supplementary figures and images for: Identification of key programmed cell death genes for predicting prognosis and treatment sensitivity in colorectal cancer
Source: Front Oncol. 2024 Nov 13;14:1483987. doi: 10.3389/fonc.2024.1483987 (PMC11603958; doi:10.3389/fonc.2024.1483987)

**Fig 10I**

**LOVO**

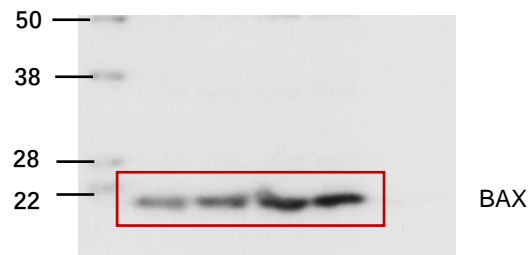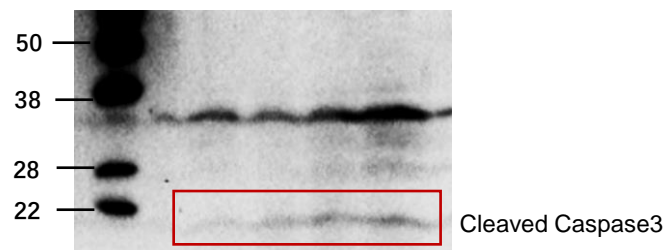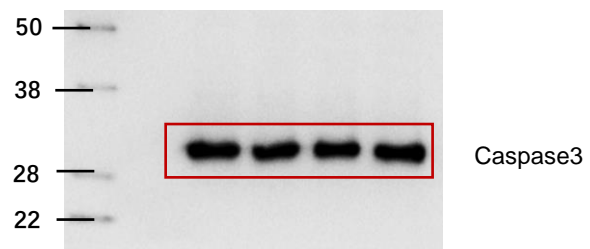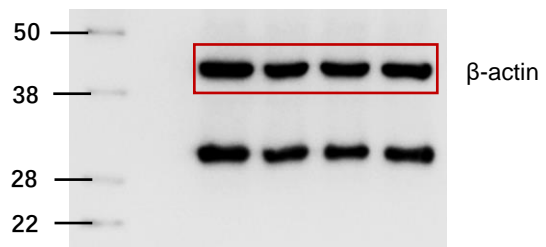

**Fig 10J**

**SW480**

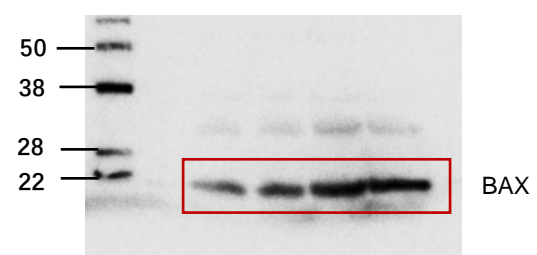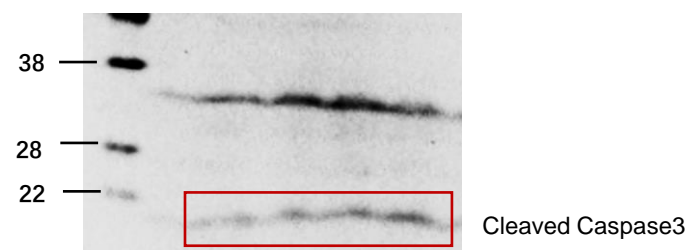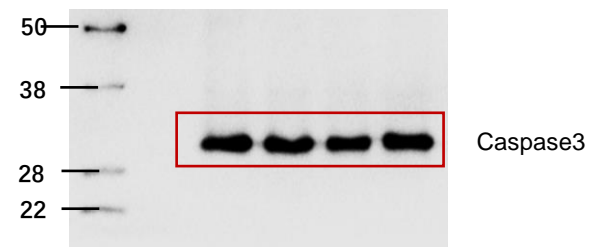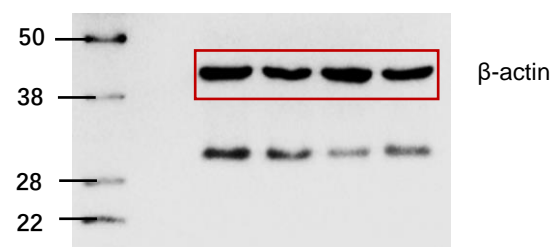

Supplement: Supplementary Figure S1 — relationship between expression levels of the three model genes and OS separately. [file DataSheet1.zip › full_membranes_WB.pdf]
